# Progress in Bacteriology

**Published:** 1900-11-03

**Authors:** 


### Progress in Bacteriology.

**Bacteriology of Gangrene of the Lung.**—Babes[1] states that the micro-organisms so frequently described as associated with gangrene of the lung, viz., leptothrix, sarcinæ, and spirochetæ, are only superadded saprophytic varieties. The organisms capable directly of exciting gangrene are highly intensified staphylo-, strepto-, and pneumo-cocci, a bacillus resembling the B. œdema maligni, and a coliform bacillus which liquifies gelatin. With these are associated saprophytic varieties, and not infrequently bacilli with the characters of the diphtheria bacillus. Varieties of similar organisms he finds are present in the hæmorrhages and in the organs of cases of tuberculosis which exhibit hæmorrhagic infection, purpuric rashes, &c. It would appear that any tubercular lesions, but more especially pulmonary cavities, are foci from which a general infection may spread, such infection may result in gangrene, hæmorrhagic septicæmia, typhoid fever, or hepatic suppuration. The two organisms most commonly met with in these secondary infections are the pseudo-diphtheria bacillus, and a bacillus resembling the typhoid bacillus.

**The Strepto-coccus.**—The same author has conducted important researches upon the strepto-cocci. He concludes that the division into long and short varieties is useless, and that each variety should be described according to its behaviour in different media, to temperature, to oxygen, and to colour formation. He describes eleven different varieties of strepto-cocci. All these are subject to seasonal alterations in virulence, and some, without localising themselves in any organ, are capable of bringing about a fatal termination.

**Diphtheroid Bacilli in Milk.**—Eyre[2] describes three organisms found in milk. All resemble the Klebs-Löffler bacillus in exhibiting segmentation, metachromatism, and clubbed involution forms. The colonies of one variety are white, of the second, yellow, and of the third a faint pink accompanied by a strong pinkish discolouration of the medium. None of these three groups are pathogenic. Microscopically they are indistinguishable from the Klebs-Löffler bacillus.

**Bacteria in Human Milk.**—Basch and Weleminsky[3] show that in animals acutely infected by cultures of enteric fever, cholera, diphtheria, and anthrax, the milk remains sterile, but that in severe and long-continued sickness, or in local lesions of the mammary gland organisms may be found in the milk. Thus a phthisical mother may infect her breast-fed child. Such a case is recorded by Roger and Garnier.[4] The milk in this case was pathogenic to guinea-pigs.

**B. Typhosus and Allied Organisms.**—Cushing[5] describes a new pathogenic bacillus which he isolated from an osteomyelitic abscess following a febrile attack resembling typhoid fever. This bacillus belonged to the Gärtner type, and was akin to the B. enteritidis, B. cholera suis, &c., intermediate between the B. typhosus and B. coli groups. Possibly many febrile attacks of typhoidal nature in which the blood of the patient does not give Widal's reaction are due to infections by members of this class, and the blood will be found to have specific agglutinating reactions. The Gärtner group presents an early and terminal strong alkalinity in milk, appearing after a transient acidity. Milk is never coagulated. Glucose is fermented, never lactose or saccharose. No indol is formed. At a time when so much attention is being paid to the biological treatment of sewage, the statement of Houston[6] that bacterial beds are of no effect in the mechanical separation of micro-organisms is one of great importance. Examination of various effluents showed that B. coli were present equally in these and in the crude sewage. Similarly the spores of B. enteritidis were detected in the effluents, nor indeed was their number diminished. There is, too, evidence that the B. typhosus can multiply in the effluent from biological sewage filters, and the more delicate organisms, such as strepto-cocci, B. tuberculosis, and B. tetani are uninjured. Remy[7] strongly opposes the idea of their being any relationship between coliform and typhoid bacilli. The Eberth-Gaffky bacillus is never found in any disease other than enteric fever, and in this disease its virulence lessens gradually after the second week. He states that by using a special gelatine medium the typhoid bacilli in stools may readily be separated from coli varieties. The medium, however, is, we think, too complex for general adoption, containing oxalic, lactic, and citric acids, sulphates of magnesium and potassium, and phosphate and chloride of sodium,

**Rheumatic Fever.**—Poynton and Paine[8] describe a diplococcus which they have found in the blood, serous effusions, and tissues, and on the tonsils of persons suffering from acute rheumatism. The diplococcus grows best in fluid media, milk, or broth, acidified with lactic acid. It may also be grown in blood agar, and is a facultative anærobe. Injected into guinea-pigs, the diplococcus excited acute arthritis of a non-suppurative character, pericarditis, endocarditis, or nephritis. It was recovered again from the tissues after death. In fluid media it forms chains. The individual elements of the diplococcus measure about $\cdot 5\mu$, and the colonies in blood agar have a yellowish-white appearance not unlike those of staphylococcus. It stains well with all aniline dyes, but is decolorised by Gram. Tissues are best prepared with carbolthionin. It is interesting to note that the organism is found in the kidney and urine of patients suffering from rheumatic fever; and the authors think that it may be the causative agent in many cases of infective endocarditis. A similar organism has been described by Triboulet and Wasserman.

**Scarlet Fever.**—Baginsky and Sommerfeld[9] describe an organism as the cause of this disease. It is polymorphous, the commonest forms being diplococcus, tetracoccus, streptococcus, and staphylococcus, according to the age of the culture and the nature of the medium. Class[10] claims that this is the same organism described by him as a large gonococcus-like body, but thinks that the streptococcus forms must have been due to accidental contamination. Class found that cultures injected intravenously into white swine gave rise to a disease characterised by a red rash, high temperature, and acute nephritis, subsequently followed by desquamation. It is found in the blood, scales, and on the tonsils of patients suffering from scarlet fever.

**A New Stain for Gonococci.**[11]—Kresylecht-violet in an aqueous solution (1 in 10,000) stains the nuclei of pus cells a faint blue, and the gonococcus red violet. Other bacteria stain with great difficulty in this solution.

**B. Ærogenes Capsulatus.**—This organism has ong been known as the exciting cause of the condition known as "foam organs," an emphysematous state of the viscera discovered after death. Welch[12] has recently given considerable additional information regarding this bacillus, which he states is entirely distinct from the bacillus of malignant œdema. The B. ærogenes capsulatus slowly liquefies gelatin, and has now been demonstrated in a sporing condition. Although the author believes gas formation to be chiefly a post-mortem occurrence, he yet inclines to the idea that it may occur during life. The B. ærogenes capsulatus is the commonest cause of emphysematous gangrene; and in some cases the wound has apparently been affected from the general circulation, and this is not unlikely as the bacillus is a normal inhabitant of the intestine. About 50 per cent. of cases die. Films should be prepared and stained by Gram's method. The B. ærogenes capsulatus is also found in gaseous abscesses, uterine infections, infections of the urinary tract, gastro-intestinal canal, and pulmonary system.

[1] Ann. de Path. et Bact. Bucharest, vol. vi. [2] Brit. Med. Jour., Aug. 18, 1900. [3] Arch. f. Hyg., vol. xxxv., p. 205. [4] Compt. Rend. de Biol., March 2, 1900. [5] Bull. Johns Hopkins Hosp., July-Aug. 1900. [6] Brit. Med. Jour., Aug. 18, 1900. [7] An. de l'Inst. Pasteur, Aug. 25, 1900. [8] Lancet, Sept. 22, 1900. [9] Ber. Klin. Woch., July 2, 1900. [10] Lancet, Sept. 29, 1900. [11] Centralb. f. Bakt., xxvii., 533, 1900. [12] Johns Hopkins Bull., Sept. 1900.

